# Transcriptome Analysis Reveals a Potential Response Mechanism of *Morchella* to Soybean Meal Addition

**DOI:** 10.3390/jof12090660

**Published:** 2026-09-02

**Authors:** Yihong Yue, Qian Wang, Yuchen Zhang, Tingting Xiao, Haibo Hao, Zongjun Tong, Wei Li, Jingjing Huang, Jinjing Zhang, Hui Chen

**Affiliations:** 1 National Engineering Research Center of Edible Fungi, Key Laboratory of Edible Fungi Resources and Utilization (South), Ministry of Agriculture, Institute of Edible Fungi, Shanghai Academy of Agricultural Sciences, Shanghai 201403, China; yihongyue@saas.sh.cn (Y.Y.); wq-15309@163.com (Q.W.); zyc0216@saas.sh.cn (Y.Z.); xiaotingting@saas.sh.cn (T.X.); hhb199232@163.com (H.H.); ttzjun1@163.com (Z.T.); 2 Amway (China) Botanical Research and Development Center, Wuxi 214145, China; wei.li@amway.com (W.L.); evelyn.huang@amway.com (J.H.)

**Keywords:** *Morchella*, soybean meal addition, transcriptome analysis, protein content, tissue specificity

## Abstract

*Morchella* is an edible and medicinal fungus valued for its distinctive flavor and nutritional properties. However, unstable yield and quality remain major constraints on its commercial cultivation. Although soybean meal is used as an organic nutrient supplement in edible mushroom production, its effects on protein accumulation and transcriptional regulation in *Morchella* remain unclear. This study combined fruiting-body protein measurements with transcriptome analysis to compare soybean meal supplied through soil application or exogenous nutrient bags and to characterize tissue-specific responses in the cap and stipe. Soybean meal increased protein content in an application method-, concentration-, and tissue-dependent manner. Soil application was more effective than nutrient-bag supplementation, while stipes exhibited a stronger transcriptional response than caps. RNA sequencing detected 11,982 expressed genes and revealed both shared and tissue-specific responses to the two supplementation methods. Gene Ontology analysis showed broadly similar functional categories among comparison groups, whereas KEGG enrichment analysis revealed distinct metabolic responses. In caps, soil application was associated with glutathione metabolism and amino sugar and nucleotide sugar metabolism, while nutrient-bag supplementation was associated with the pentose phosphate pathway, glyoxylate and dicarboxylate metabolism, and pentose and glucuronate interconversions. In stipes, the genes involved in ribosome and aminoacyl-tRNA biosynthesis were downregulated under soil application and nutrient-bag supplementation, respectively. Weighted gene co-expression network analysis identified three modules associated with protein content and highlighted ribosome, aminoacyl-tRNA biosynthesis, glycerophospholipid metabolism, and arginine biosynthesis as candidate pathways. The most highly connected genes in each key module were identified as hub genes, and selected expression patterns were supported by qRT-PCR. These findings provide a transcriptomic framework for understanding how soybean meal supplementation is associated with protein accumulation and tissue-specific physiological responses in *Morchella*.

## 1. Introduction

Morels (*Morchella* spp.) belong to the family *Morchellaceae*, order Pezizales, class Pezizomycetes, and phylum Ascomycota, and are among the few macroascomycetes that can be cultivated artificially. *Morchella* is an edible and medicinal fungus valued for its unique flavor, high nutritional value, and medicinal properties and is widely distributed worldwide [1,2]. From a nutritional perspective, *Morchella* is rich in protein, with 24.5 g/100 g protein and 7.7–56.9 mg/g amino acids, including a variety of essential amino acids [3,4,5]. As the global population grows and climate change intensifies, the supply of animal-derived foods may be unable to meet increasing demand [6]. Therefore, alternative sources of high-quality protein are urgently needed, and *Morchella* may represent a novel protein source and a potential alternative to animal-derived protein.

In recent years, artificial cultivation technology for *Morchella*, characterized by exogenous nutrient bags, has achieved significant breakthroughs in China, which provides critical and sustainable organic nutrients, enabling *Morchella* to complete its life cycle in artificial environments [7,8]. Substrate serves as the most critical part of mushroom development, and its nutrient supply mode directly affects the growth process of *Morchella*. The nutrients required for *Morchella* growth are derived mainly from exogenous nutrient bags and soil. Previous studies revealed that exogenous nutrient bags primarily provide a carbon source for *Morchella* fructification, while the essential nitrogen required for ascocarp formation seems to be highly dependent on the supply from the soil substratum [9]. As a key nutritional factor affecting the growth and development of edible fungi, nitrogen sources in the substratum are essential for mycelial growth, fruiting body development, and yield formation [10,11]. However, most lignocellulosic cultivation materials, such as corncobs, crop straw, and cottonseed hulls, have low nitrogen contents (<1%); therefore, additional nitrogen sources may be required for edible mushroom cultivation [12,13,14,15,16,17]. These supplements include complex organic nitrogen sources, such as bran, husk, and soybean meal; chemically simple organic nitrogen sources, such as urea [CO(NH_2_)_2_]; and inorganic nitrogen sources, such as ammonium chloride. Although urea is a naturally occurring organic compound, commercially available urea is generally manufactured synthetically and represents a chemically simple nitrogen source [18]. In contrast, soybean meal, a byproduct of soybean oil extraction, is a complex organic nitrogen source containing 40–49% crude protein, together with peptides and various amino acids, and has a total nitrogen content of 7.77–8.00% [19,20]. The diverse organic nutrients supplied by soybean meal can effectively meet the nitrogen requirements of fungal growth [21]. Some studies have shown that the addition of soybean meal to the cultivation substrates of *Pleurotus eryngii* var. *eryngii* [22], *Hericium erinaceus* [23], and *Pleurotus sajor-caju* [24] can significantly increase the quantity and quality of their fruiting bodies, with particularly pronounced effects in species with high nutritional demands [25]. Although the ability of soybean meal to promote the growth of edible fungi has been partially confirmed, the intrinsic mechanism through which it regulates protein synthesis in the cap and stipe of *Morchella* at the molecular level remains unclear.

With the expansion of commercial cultivation, unstable yield and quality have become major constraints on the development of the *Morchella* industry [26,27], partly because the mechanisms underlying its responses to nutrient supplementation remain insufficiently understood. We hypothesized that soybean meal supplementation would alter protein accumulation in *Morchella* fruiting bodies by affecting transcriptional processes associated with nitrogen utilization, protein synthesis, and carbon metabolism, and that the magnitude and molecular characteristics of this response would differ between application methods and between the cap and stipe. This hypothesis was evaluated by measuring the protein contents of caps and stipes under soil and nutrient-bag supplementation and by characterizing their transcriptional responses through differential expression analysis, functional enrichment analysis, weighted gene co-expression network analysis, and qRT-PCR validation. The specific objectives were to (i) determine the effects of soybean meal application method and dose on the protein contents of *Morchella* caps and stipes; (ii) characterize the shared and tissue- or application-specific differentially expressed genes and enriched pathways; and (iii) identify protein-content-associated co-expression modules and candidate hub genes. This study provides a molecular basis for optimizing soybean meal supplementation in *Morchella* cultivation and improving fruiting-body protein content.

## 2. Materials and Methods

### 2.1. Fungal Collection and Experimental Treatment

The cultivated strain of *Morchella sextelata*, Shenyang No. 4, was used in this study and was preserved at the Institute of Edible Fungi, Shanghai Academy of Agricultural Sciences (Shanghai, China). To investigate the effects of soybean meal supplementation on the protein content of *Morchella*, five experimental groups were established: an untreated control without soybean meal supplementation (CK), two soil-application treatments receiving 100 or 1000 g/m^2^ soybean meal, and two nutrient-bag treatments containing 1% or 10% soybean meal. Soil application and nutrient-bag supplementation were conducted as separate treatment modes, and no treatment received soybean meal through both routes. Each treatment was established in five replicate field plots, and identical cultivation practices were applied across all treatments. At the designated sampling stage, three field plots were independently selected from each treatment. Five fruiting bodies were collected from each selected plot. After roots and adhering debris were removed, the caps and stipes were separated using a sterile scalpel. The five caps and five stipes obtained from the same plot were pooled separately to form one cap biological replicate and one stipe biological replicate, respectively. Thus, three independent biological replicates were obtained for each tissue under each treatment, resulting in a total of 30 tissue samples (5 treatments × 2 tissues × 3 biological replicates). All samples were immediately immersed in liquid nitrogen and stored at −80 °C until further analysis.

### 2.2. RNA Extraction, Library Preparation, and Illumina Sequencing

Eighteen fruiting-body tissue samples representing six experimental groups were selected for transcriptome sequencing, with three biological replicates per group. CCK and SCK represent the cap and stipe, respectively, from the untreated control group; CA and SA represent the cap and stipe, respectively, from fruiting bodies receiving 100 g/m^2^ soybean meal through soil application; and CS and SS represent the cap and stipe, respectively, from fruiting bodies receiving 1% soybean meal through nutrient-bag supplementation. Total RNA was extracted using TRIzol^®^ Reagent (Invitrogen, Carlsbad, CA, USA) according to the manufacturer’s instructions. RNA quantity was measured using an Invitrogen Qubit 4.0 fluorometer (Thermo Scientific, Waltham, MA, USA), and RNA quality was further checked using an Agilent 2100 Bioanalyzer (Agilent Technologies, Santa Clara, CA, USA) with OD260/280 = 1.8–2.2, OD260/230 ≥ 2.0, RIN ≥ 6.5, 28S:18S ≥ 1.0, and a total RNA amount > 10 μg. Sequencing libraries were prepared using a TruSeq™ RNA sample preparation kit (Illumina, San Diego, CA, USA) following the manufacturer’s instructions. The generated libraries were subsequently sequenced on the Illumina NovaSeq 6000 platform (Illumina, San Diego, CA, USA) with a paired-end run (2 × 150 bp) by Shanghai Majorbio Co., Ltd. (Shanghai, China).

### 2.3. Differentially Expressed Gene (DEG) Analysis

The raw paired-end reads were trimmed and quality controlled using SeqPrep (https://github.com/jstjohn/SeqPrep, accessed on 28 August 2026) and Sickle (https://github.com/najoshi/sickle, accessed on 28 August 2026), respectively, with default parameters. The clean reads were subsequently aligned to the *M. sextelata* genome ASM2013738v1 (https://www.ncbi.nlm.nih.gov/datasets/genome/GCF_020137385.1/, accessed on 24 September 2021) in orientation mode using Hierarchical Indexing for Spliced Alignment of Transcripts (HISAT2, v2.0.5, https://ccb.jhu.edu/software/hisat2/index.shtml, accessed on 28 August 2026) software [28]. The mapped reads of each sample were assembled via a reference-based approach by StringTie (v1.3.3b, https://ccb.jhu.edu/software/stringtie/index.shtml?t=example, accessed on 28 August 2026) [29]. To identify differentially expressed genes (DEGs), the expression of each transcript was calculated using the transcripts per million reads (TPM) method, which considers both the gene length and the number of mapped reads. RNA-seq by expectation–maximization (RSEM, https://github.com/deweylab/RSEM, accessed on 28 August 2026) was employed to quantify gene abundances [30]. Differential expression analysis between experimental groups was performed using the DESeq2 R package v1.16.1 [31]. Genes were considered differentially expressed based on the following criteria: |log2 fold change (log2FC)| > 1 and q < 0.05. Principal component analysis (PCA) was performed using the prcomp function in R(v4.3.2) based on log2(TPM + 1)-transformed expression values of all expressed genes. The expression data were centered and scaled before PCA, and the first two principal components were used to evaluate sample clustering and separation among the experimental groups. Volcano plots were generated using the ggplot2 package in R, with log2FC on the *x*-axis and −log10(q value) on the *y*-axis. Genes meeting both |log2FC| > 1 and q value < 0.05 were defined as DEGs. DEGs with log2FC > 1 were classified as upregulated, whereas those with log2FC < −1 were classified as downregulated; genes that did not meet the DEG criteria were classified as non-DEGs. For the UpSet analysis, gene IDs from the four DEG comparisons (CA vs. CCK, CS vs. CCK, SA vs. SCK, and SS vs. SCK) were treated as four sets, irrespective of the direction of differential expression. All 15 non-empty exclusive intersections were calculated using a custom Python (v3.11.9) script and visualized using Matplotlib (v3.8.4). Each gene was counted once in the exclusive intersection corresponding to its exact membership pattern.

### 2.4. GO and KEGG Pathway Enrichment Analyses

Gene functional annotation was performed through the Gene Ontology (GO, http://geneontology.org/) database and the Kyoto Encyclopedia of Genes and Genomes (KEGG, http://www.genome.jp/kegg/) database using the phyper package in R software. The identified DEGs were further subjected to GO functional enrichment and KEGG pathway analysis, which were executed via Goatools (v0.9.9, https://github.com/tanghaibao/Goatools, accessed on 28 August 2026) and KOBAS (v3, http://bioinfo.org/kobas, accessed on 28 August 2026) [32], respectively.

### 2.5. Weighted Gene Co-Expression Network Analysis (WGCNA)

Weighted gene co-expression network analysis (WGCNA) was performed across all RNA-seq samples using the WGCNA package (v1.72-5) in R to identify co-expression modules associated with protein content and candidate hub genes. The soft-thresholding power (β) was selected according to the scale-free topology criterion while retaining adequate mean network connectivity. The minimum module size was set to 30, and modules with similar expression profiles were merged using a cut height of 0.25. Genes with similar expression patterns were grouped into the same module through correlation analysis and hierarchical clustering, and each module was assigned a unique color. The module eigengene was calculated as the first principal component of the expression profiles of all genes within a module and was correlated with protein content to identify key modules. KEGG pathway enrichment analysis was subsequently performed for genes in the key modules, with a *p* value < 0.05 used as the enrichment threshold.

For network visualization, genes assigned to significantly enriched pathways in each key module were visualized using Cytoscape (v3.7.2). Because WGCNA was performed across all samples rather than separately for each treatment, treatment-specific network topologies were not constructed. The Connectivity value obtained from the WGCNA network output was used to assess the relative topological importance of each gene within the corresponding key-module network. Larger Connectivity values indicate stronger overall connections between a gene and the other genes in the network. Genes were ranked according to their Connectivity values, and the most highly connected genes were designated as hub genes.

### 2.6. Quantitative Real-Time PCR (qRT-PCR)

To validate the RNA-seq data, 12 DEGs were randomly selected from the integrated pathway analysis, including six genes from the cap and six from the stipe. Primer sequences are listed in Table S1. The *actin* gene of *M. sextelata* served as the internal reference, and the corresponding untreated cap or stipe sample was used as the calibrator. Three independent biological replicates were analyzed for each treatment. Relative gene expression was calculated using the 2^−ΔΔCt^ algorithm.

### 2.7. Statistical Analysis

The biochemical analysis and the RNA-Seq assay were performed in triplicate for each treatment. The bar graphs were drawn and statistically evaluated using GraphPad Prism v9.5.1 (GraphPad Software, Boston, MA, USA). One-way analysis of variance (ANOVA) and Duncan’s multiple range test were used to detect the significance using the IBM SPSS 25.0 statistical package (IBM Inc., Armonk, NY, USA). Differences with *p*  <  0.05 were considered statistically significant. All datasets were expressed as the mean ± standard error of mean (SEM).

## 3. Results

### 3.1. Effects of Soybean Meal Supplementation on Fruiting-Body Protein Content

**Figure 1:**
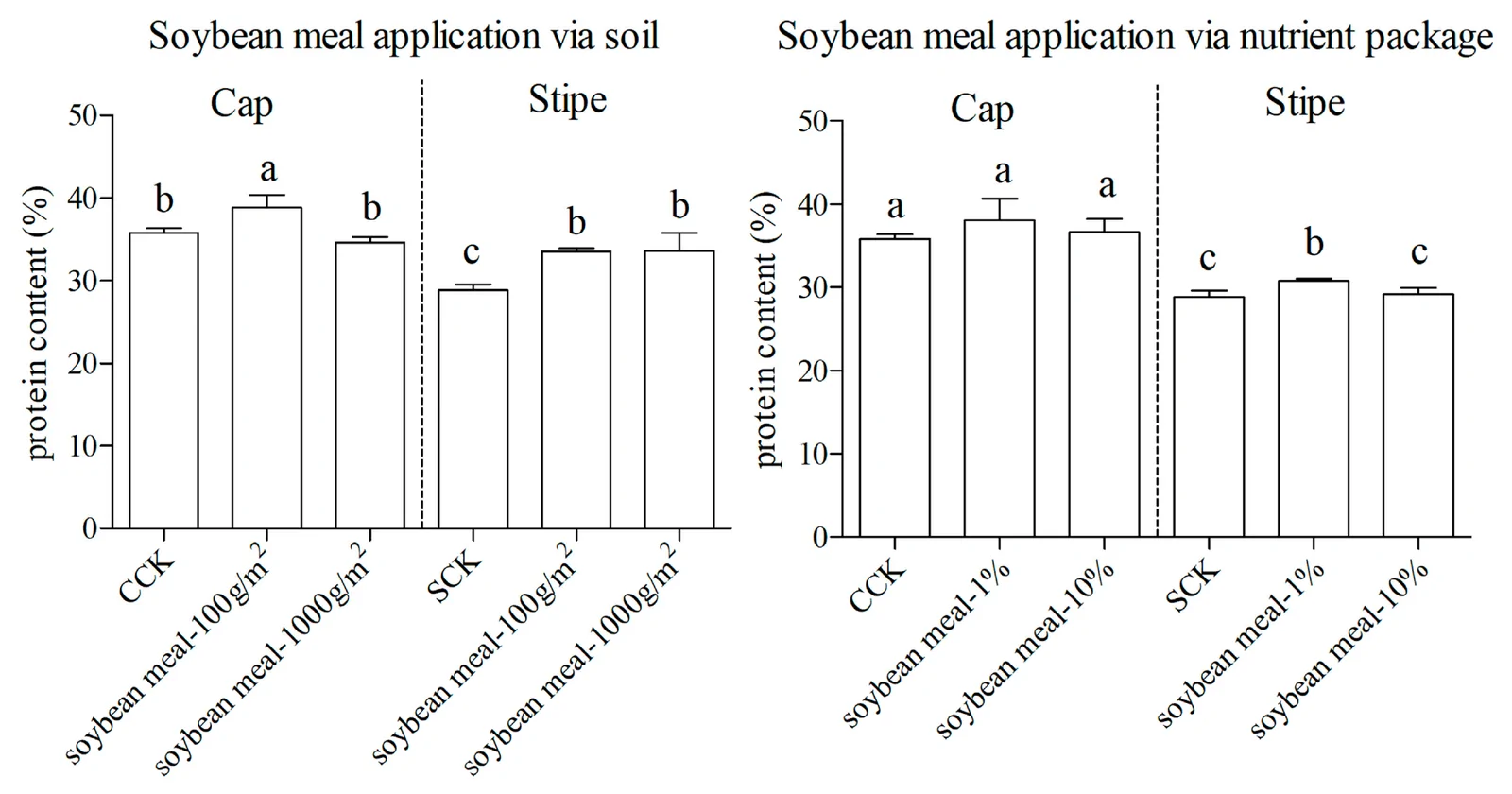
Effects of soil application and nutrient-bag supplementation with soybean meal on the protein contents of the cap and stipe tissues of *Morchella* fruiting bodies. Different lowercase letters indicate significant differences among treatments within the same tissue (Duncan’s multiple range test, *p* < 0.05). Soil application and nutrient-bag supplementation were conducted as separate treatment modes; soybean meal was not applied through both routes within the same treatment.

After soybean meal was applied through soil application and nutrient-bag supplementation, the protein contents of cap and stipe tissues from mature fruiting bodies were analyzed separately. As shown in Figure 1, under soil application, the 100 g/m^2^ soybean meal treatment significantly increased the protein content of the caps, reaching an average value of 388.65 g/kg, which was 8.50% greater than that of the CCK group (*p* < 0.05). In contrast, the protein content of the caps in the 1000 g/m^2^ soybean meal treatment was 346.55 g/kg, which was not significantly different from that in the CCK group (*p* > 0.05). In stipe tissues, compared with the SCK treatment, both the 100 g/m^2^ and 1000 g/m^2^ soybean meal treatments significantly increased the protein content (*p* < 0.05), with increases of more than 16.61%; however, no significant difference was observed between the two soybean meal concentrations (*p* > 0.05). Under nutrient-bag supplementation, the 1% soybean meal treatment resulted in the highest protein content in the caps, with an average value of 380.70 g/kg, representing a 6.28% increase compared with that in the CCK group. Compared with the CCK treatment, the 10% soybean meal treatment increased the cap protein content by 2.20%. In the stipes, the 1% soybean meal treatment resulted in the highest protein content, with an average value of 307.35 g/kg, which was 6.75% greater than that of the SCK group and significantly greater than those of the SCK and 10% soybean meal treatments (*p* < 0.05). In contrast, compared with the SCK treatment, the 10% soybean meal treatment increased stipe protein content by only 1.19%, which was not significantly different from that in the SCK group (*p* > 0.05). Moreover, under both soybean meal application methods, the protein content of the caps was significantly greater than that of the stipes (*p* < 0.05), indicating clear tissue-specific differences in protein accumulation in *Morchella* fruiting bodies. Overall, the effects of soybean meal addition on protein accumulation in *Morchella* fruiting bodies were associated with the application method, application concentration, and tissue type. Specifically, the 100 g/m^2^ soil application treatment had the most pronounced effect on increasing the cap protein content, whereas the 1% nutrient bag supplementation treatment had the strongest effect on increasing the stipe protein content.

### 3.2. Transcriptome Analysis

To better investigate the molecular mechanism underlying the increased protein content in response to soybean meal addition in *M. sextelata*, 18 samples from the CK and soybean meal treatment groups were subjected to transcriptome sequencing on the Illumina platform. Samples were selected as follows: CCK and SCK (caps and stipes from the CK group), CA and SA (caps and stipes from fruiting bodies treated with 100 g/m^2^ soybean meal through soil application), and CS and SS (caps and stipes from fruiting bodies treated with 1% soybean meal through nutrient-bag supplementation). A statistical overview of the sequencing results is shown in Tables S2 and S3. After purification and filtration, a total of 43.97–47.36 million clean reads per sample were generated, accounting for approximately 99.02–99.28% of the raw reads (Table S2). More than 6.02 Gb of clean data were obtained for each sample, with the Q20 and Q30 percentages exceeding 98.39% and 94.66%, respectively, and the sequencing error rate was less than 0.013%. All the libraries presented GC contents greater than 49.24%. In terms of mapping efficiency, the total number of mapped reads across the libraries ranged from 89.49% to 95.54%, with multiple-mapped reads comprising 2.06–3.40% of the total number of mapped reads (Table S3). Notably, 86.92–93.14% of the reads were uniquely mapped to the M. sextelata reference genome, while the expression profiles of 11,982 genes were determined through the quantification of transcriptomic abundance utilizing transcripts per million (TPM) values. These results indicated that the quality of the transcriptome sequencing data was good and could be used for subsequent analysis.

### 3.3. Analysis of Differentially Expressed Genes

**Figure 2:**
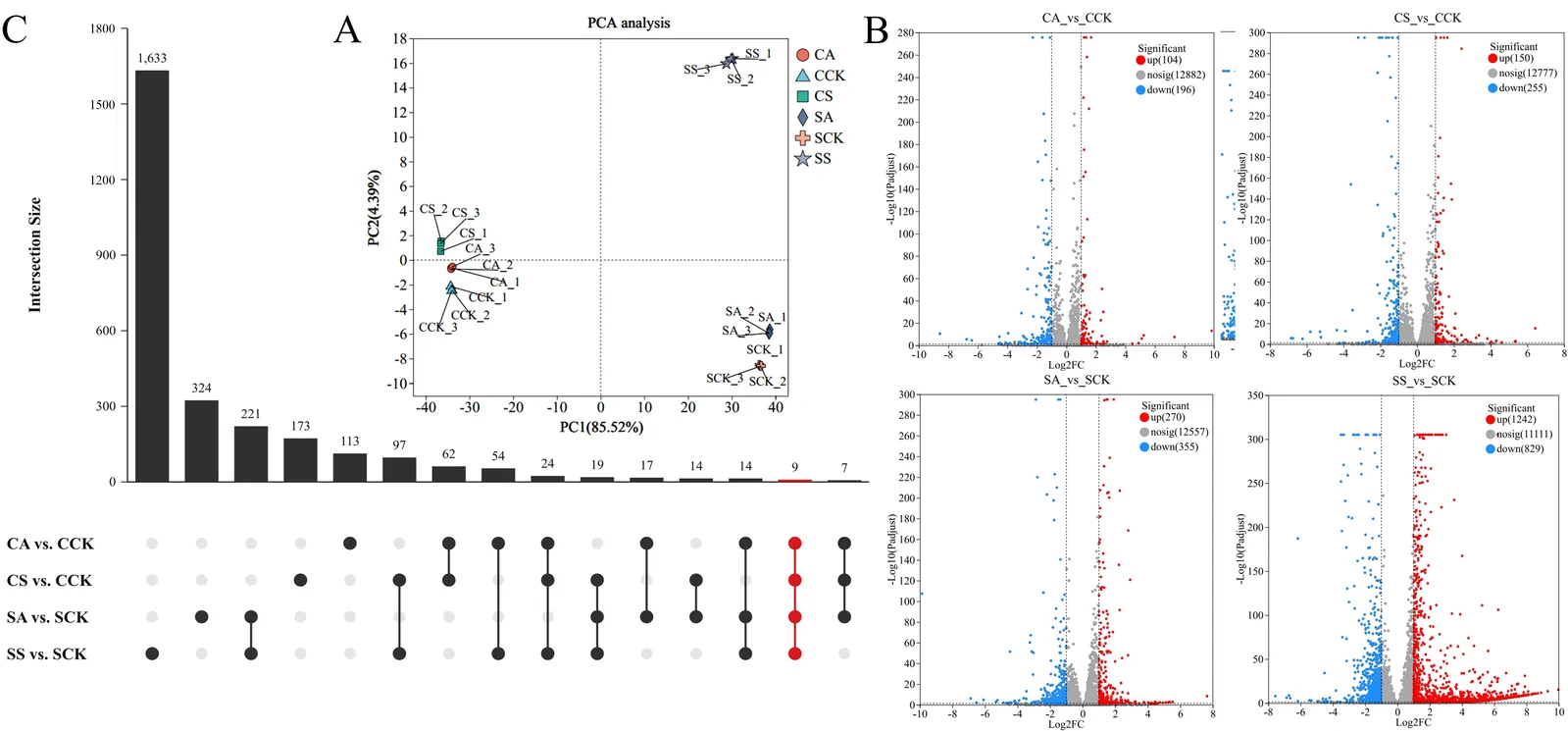
Principal component analysis and differential gene-expression patterns in the cap and stipe tissues of *M. sextelata* under different soybean meal treatments. (**A**) Principal component analysis (PCA) of the 18 RNA-seq samples. (**B**) Volcano plots of differentially expressed genes (DEGs) in the CA vs. CCK, CS vs. CCK, SA vs. SCK, and SS vs. SCK comparisons. Genes with |log2FC| > 1 and q value < 0.05 were defined as DEGs. Red, blue, and gray points represent upregulated DEGs, downregulated DEGs, and non-DEGs, respectively. (**C**) UpSet plot showing the sizes of the DEG sets and their exclusive intersections across the four comparisons. The vertical bars indicate the number of genes in each exclusive intersection, and the connected dots indicate the comparisons included in the corresponding intersection.

Principal component analysis (PCA) revealed separation among the six experimental groups (Figure 2A). The cap and stipe samples receiving nutrient-bag supplementation showed greater separation from their corresponding controls than those receiving soil supplementation. Moreover, marked differences were also observed between the cap and stipe. These patterns indicate that the transcriptional responses to soybean meal supplementation varied with application method and tissue type.

To explore the basic regulatory patterns of *M. sextelata* in response to soybean meal addition, the DEGs identified were analyzed to determine the transcriptional level differences between various soybean meal treatments (Figure 2B). In the cap tissue, compared with the CCK group, only 300 genes (2.28% of the total gene repertoire) were significantly differentially expressed, including 104 upregulated genes and 196 downregulated genes (|log2FC| > 1, *p* < 0.05). In the CS treatment group, 405 DEGs (3.07% of the total gene repertoire) were identified, comprising 150 upregulated genes and 255 downregulated genes (|log2FC| > 1, *p* < 0.05). In the stipe tissue, compared with the SCK group, the SA treatment group contained 625 DEGs (4.74% of the total gene repertoire), 270 of which were significantly upregulated and 355 of which were significantly downregulated (|log2FC| > 1, *p* < 0.05). Compared with those in the other comparison groups, the numbers of DEGs in the SS and SCK groups were much greater. Specifically, a total of 2071 DEGs accounting for 15.71% of the total gene repertoire were detected in the SS treatment group, among which 1242 genes were upregulated and 829 genes were downregulated (|log2FC| > 1, *p* < 0.05). UpSet analysis identified 2781 unique DEGs across the four comparisons and 15 non-empty exclusive intersections (Figure 2C). Most DEGs were comparison-specific: 1633 were unique to SS vs. SCK, 324 to SA vs. SCK, 173 to CS vs. CCK, and 113 to CA vs. CCK. The largest shared intersection consisted of 221 genes detected exclusively in both SA vs. SCK and SS vs. SCK, representing a shared response of the stipe to the two soybean meal application methods. In addition, 97 genes were shared exclusively between CS vs. CCK and SS vs. SCK, whereas 62 genes were shared exclusively between CA vs. CCK and CS vs. CCK. Nine DEGs were common to all four comparisons. These results indicate that the transcriptional response to soybean meal supplementation was predominantly comparison-specific, with a smaller number of responses shared between tissues or application methods. Overall, more DEGs were detected in the stipe than in the cap, with the largest number occurring in SS vs. SCK.

### 3.4. GO Annotation Analysis of DEGs

**Figure 3:**
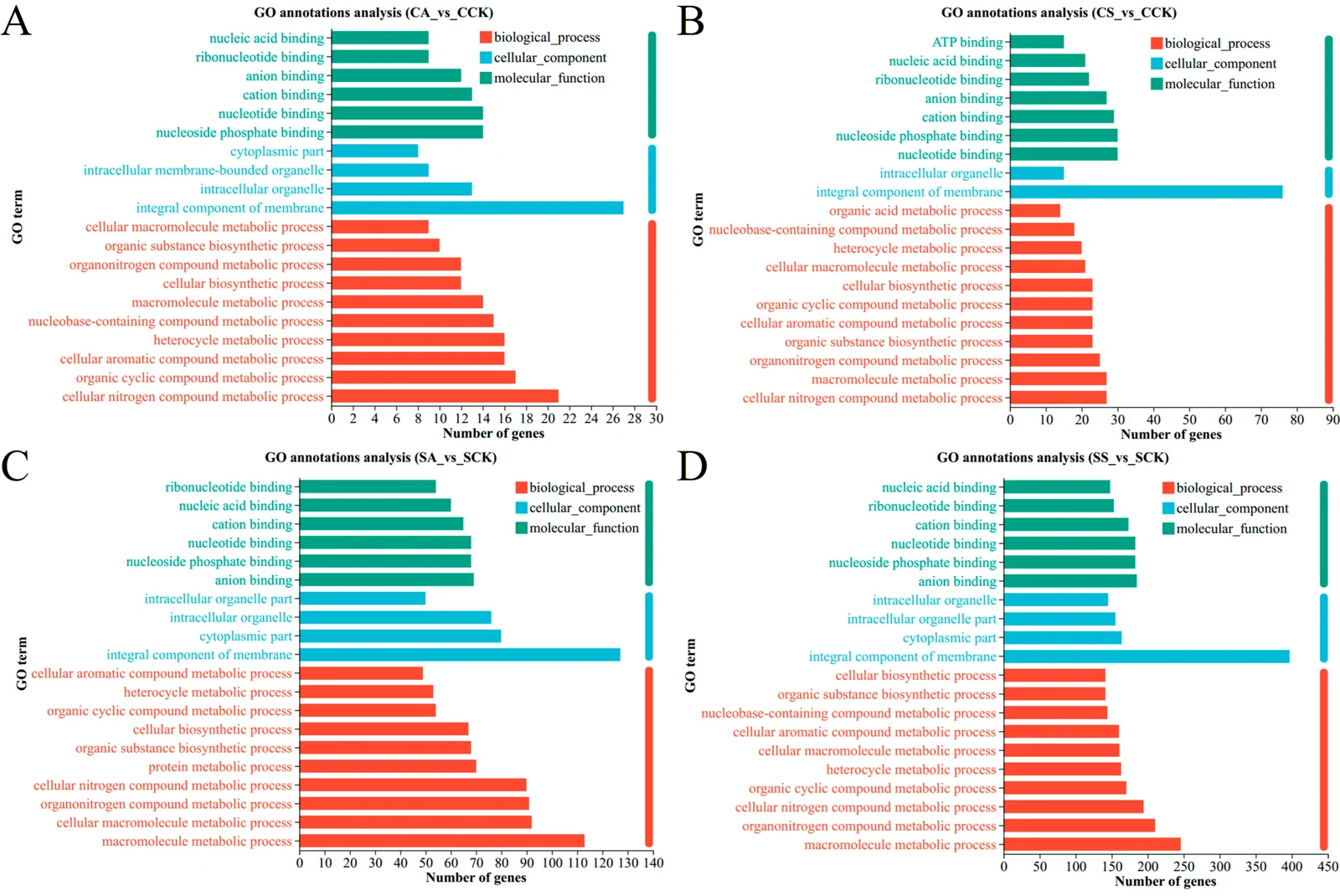
Gene classification was based on Gene Ontology (GO) annotation analysis for differentially expressed genes (DEGs) in CA vs. CCK (**A**), CS vs. CCK (**B**), SA vs. SCK (**C**), and SS vs. SCK (**D**). Different classes are shown for biological processes, cellular components, and molecular functions.

Gene Ontology (GO) annotation analysis was carried out to elucidate the functions of the DEGs involved in protein synthesis in *Morchella*. All the well-annotated DEGs were clustered into biological processes, cellular components, and molecular functions. In the comparisons of the CA vs. CCK, CS vs. CCK, SA vs. SCK, and SS vs. SCK treatments, 300, 405, 625 and 2071 genes, respectively, were annotated into the GO database among the DEGs, and assigned 123, 166, 304, and 470 GO terms, respectively. The top 20 GO enrichment terms for abundance in each comparison group are shown in Figure 3. In terms of biological processes, the DEGs in all the comparison groups were significantly enriched in metabolic processes, including the cellular nitrogen compound metabolic process (GO: 0034641), nucleobase-containing compound metabolic process (GO: 0006139), cellular aromatic compound metabolic process (GO: 0006725) and heterocycle metabolic process (GO: 0046483). In terms of cellular components, the DEGs in each treatment group were significantly enriched in cellular components related to integral component of membrane (GO: 0016021) and intracellular membrane-bounded organelle (GO: 0043231). With respect to molecular functions, the DEGs in each treatment group were significantly enriched in molecular binding functions such as nucleoside phosphate binding (GO: 1901265), nucleotide binding (GO: 0000166) and ribonucleotide binding (GO: 0032553). These results indicated that soybean meal could activate complex nitrogen metabolic networks in *Morchella*, providing precursor substances for protein synthesis by promoting the biosynthesis of nucleotides, amino acids, and their derivatives. Moreover, soybean meal stimulation might drive cells in the cap and stipe to strengthen membrane system functions, optimize transmembrane substance transport efficiency, and promote organelle differentiation to adapt to the metabolic load caused by increased nitrogen metabolic flux. Additionally, the enrichment of ATP binding (GO: 0005524) and organic acid metabolic process (GO: 0006082) was significantly greater in the CS vs. CCK comparison, indicating that nutrient release from soybean meal in nutrient packages could promote ATP production in energy metabolic pathways and accelerate the synthesis of organic acids, providing necessary energy substrates and carbon skeletons for protein synthesis.

### 3.5. KEGG Enrichment Analysis of DEGs

**Figure 4:**
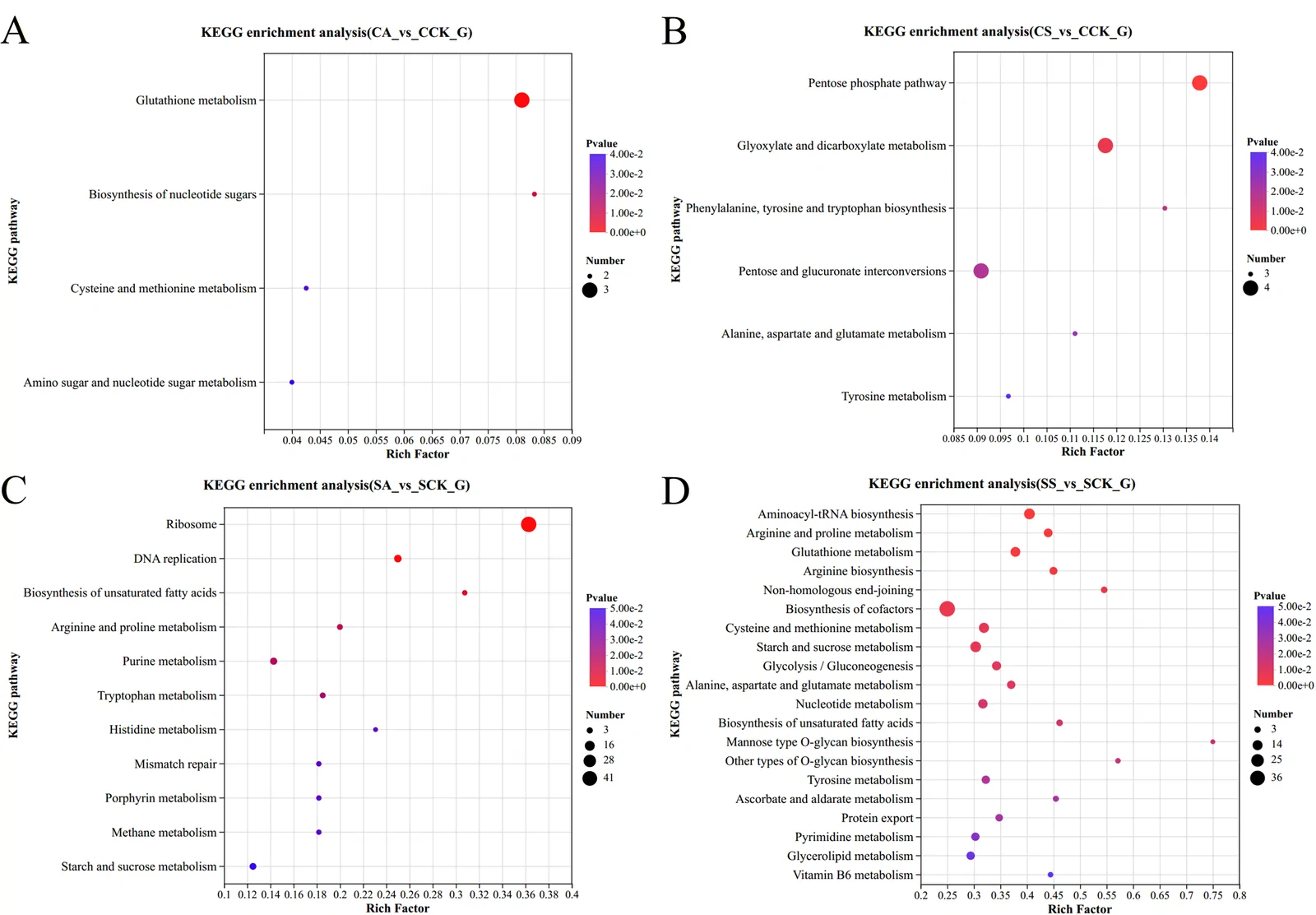
Enrichment of differentially expressed genes (DEGs) in the Kyoto Encyclopedia of Genes and Genomes (KEGG) pathways in the CA vs. CCK (**A**), CS vs. CCK (**B**), SA vs. SCK (**C**), and SS vs. SCK (**D**) comparisons.

KEGG enrichment analysis was performed to identify metabolic pathways represented among the DEGs in *Morchella* (Figure 4). In the cap, the genes *GCLM*, *GST*, and *gst*, which were assigned to the glutathione metabolism pathway, showed increased expression in CA vs. CCK (Figure 4A), suggesting an enhanced transcriptional response related to glutathione metabolism and redox homeostasis. DEGs were also associated with cysteine and methionine metabolism, which supplies sulfur-containing amino acid precursors for glutathione biosynthesis. In addition, the increased expression of *UGP2*, *galU*, *galF*, *manA*, and *MPI* in amino sugar and nucleotide sugar metabolism indicated that carbohydrate-associated processes responded to soil-applied soybean meal and may contribute carbon intermediates and reducing equivalents required for cellular metabolism and protein accumulation. In CS vs. CCK, *gnl* and *RGN*, which were assigned to the pentose phosphate pathway, showed increased expression, suggesting enhanced transcriptional activity associated with NADPH generation and pentose metabolism (Figure 4B). The increased expression of *ACAT*, *atoB*, and *MDH2*, together with the decreased expression of *FDH* and *HOGA1* in glyoxylate and dicarboxylate metabolism, indicated a reorganization of carbon-metabolism-related transcription. Furthermore, the increased expression of *UGP2*/*galU*, *galF*, and *LARA* and the decreased expression of *pel* and *ARD1* in pentose and glucuronate interconversions suggested that soybean meal supplementation affected pathways associated with polysaccharide metabolism and cell wall-related processes.

In the stipe, 41 genes encoding ribosomal proteins were downregulated in SA vs. SCK (Figure 4C), indicating that soil-applied soybean meal was associated with reduced expression of ribosome-related genes. In addition, eight genes assigned to the DNA replication pathway were downregulated, suggesting a concurrent reduction in the transcriptional activity of DNA replication-related processes. These transcriptional changes alone do not establish negative-feedback regulation or preferential utilization of exogenous amino acids. In SS vs. SCK, genes encoding aminoacyl-tRNA synthetases associated with Leu, His, Phe, Tyr, Trp, Glu, Gln, Ala, Asn, Cys, and Met were downregulated (Figure 4D), indicating that nutrient-bag supplementation altered the transcriptional regulation of aminoacyl-tRNA biosynthesis. However, the present transcriptomic data do not directly demonstrate the uptake or preferential utilization of soybean meal-derived amino acids. With respect to carbon metabolism, nutrient-bag supplementation increased the expression of genes involved in sugar-nucleotide metabolism (*UGP2*, *galU*, and *galF*) and glycogen synthesis (*GYS*, *GBE1*, and *glgB*) within the starch and sucrose metabolism pathway. This response was accompanied by decreased expression of genes encoding starch- and sucrose-degrading enzymes (*AMY*, *amyA*, and *malS*) and cellulolytic enzymes (*CBH1*, *CBH2*, and *cbhA*), together with increased expression of glycolysis-associated genes (*galM*, *TPI*, *PGK*, and *ENO*) and decreased expression of the gluconeogenesis-associated gene *FBP*. Collectively, these results indicate that soybean meal supplementation induced tissue- and application-dependent transcriptional changes in amino acid-related, translation-related, and carbon metabolic pathways.

### 3.6. WGCNA of the Transcriptome of the Morchella Fruiting Body Under Different Treatments

**Figure 5:**
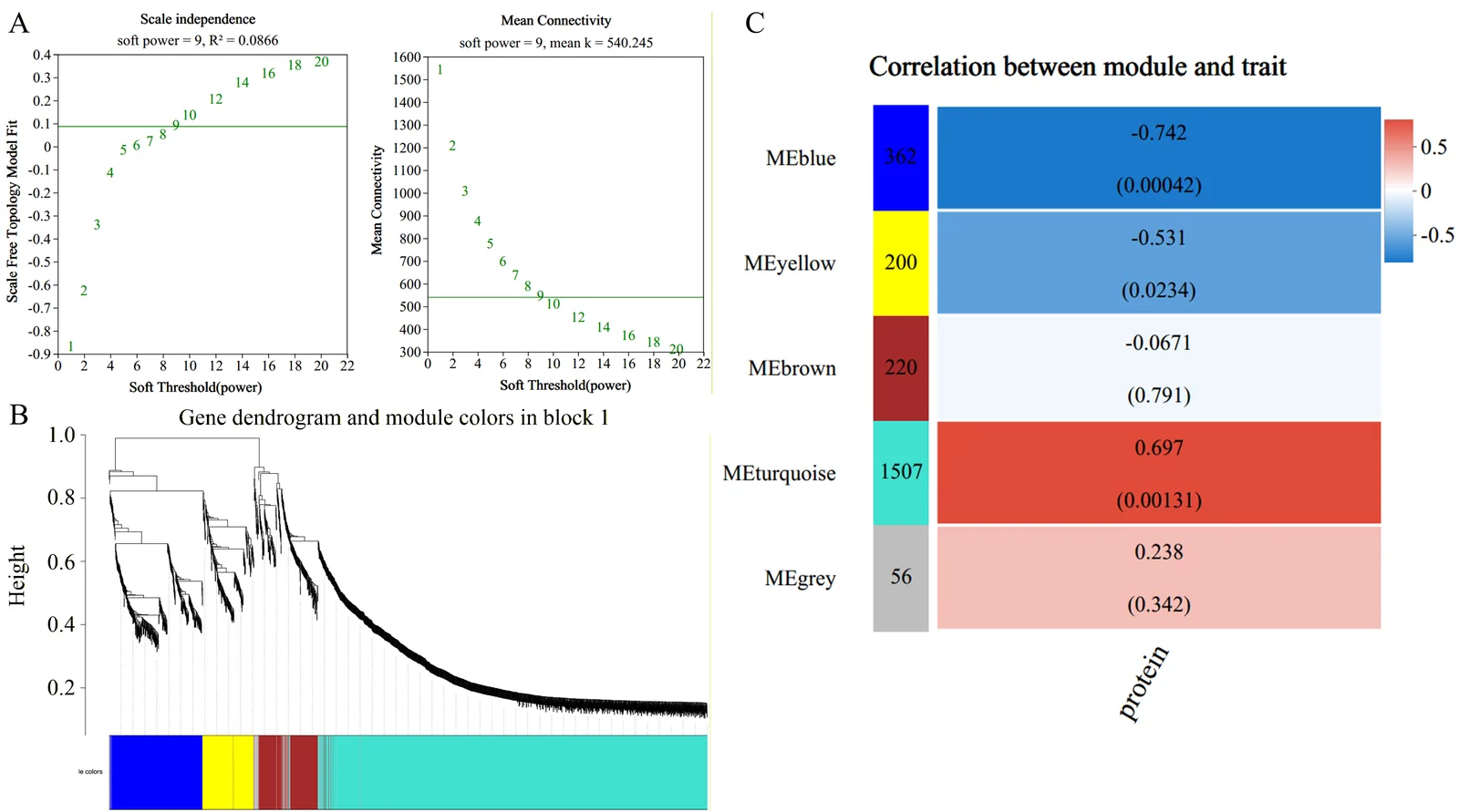
Construction of the coexpression network by weighted gene coexpression network analysis (WGCNA). (**A**) Soft power curve; the abscissa represents the power value, and the ordinate represents the correlation coefficient and the average connectivity of genes. (**B**) Hierarchical clusters represent distinct modules with coexpressed genes. Each leaflet in the clustering tree represents an individual gene. (**C**) Spearman correlation-based module trait relationships. The color grid from blue to red represents the r2 value (−1 to 1).

Weighted gene co-expression network analysis (WGCNA) was performed to identify co-expression modules associated with the protein content of *M. sextelata* (Figure 5). An adjacency matrix was constructed with a soft thresholding value of 9 to ensure that the average connectivity of the network was smooth (Figure 5A). A height cutoff of 0.25 was employed to combine similar modules, resulting in the identification of 5 unique module eigengenes (Figure 5B). The number of genes contained in each module is shown in Figure 5C, in which the blue, yellow, brown, turquoise, and gray modules contained 362, 200, 220, 1507 and 56 genes, respectively. Correlation analysis between modules and traits revealed a strong positive correlation between the turquoise module and protein content with a correlation coefficient of 0.697, whereas the blue and yellow modules displayed significant negative correlations with protein content, with correlation coefficients of −0.742 and −0.531, respectively. These results suggested that genes within the turquoise, blue and yellow modules played critical roles in the protein variation in *M. sextelata* in response to the addition of soybean meal, indicating that these three coexpression modules are key modules for further investigation.

**Figure 6:**
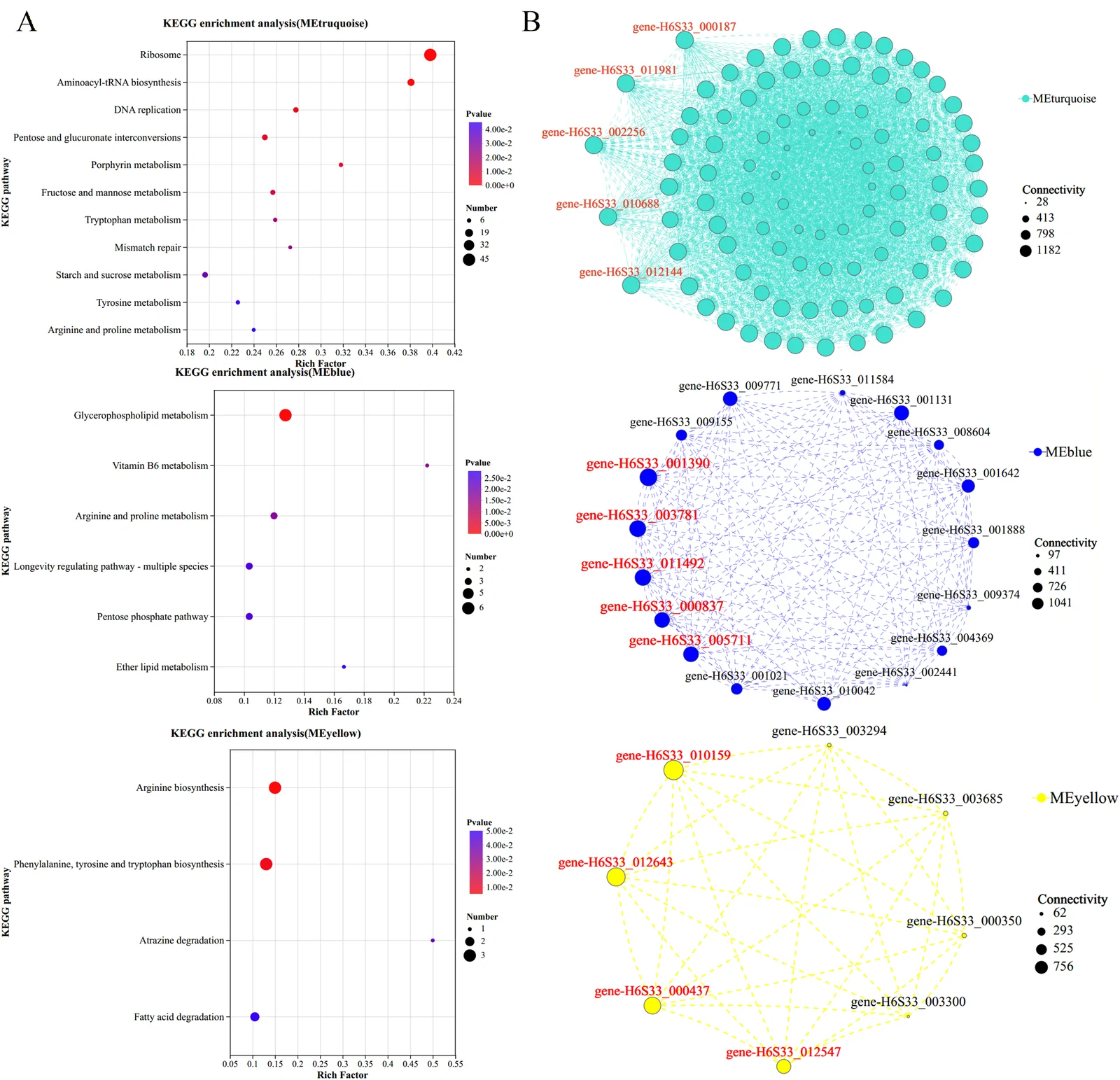
Bubble diagram of Kyoto Encyclopedia of Genes and Genomes (KEGG) pathway enrichment (**A**) and regulatory network diagram of hub genes and associated genes (**B**) in key pathways in the turquoise, blue, and yellow modules. Pathways meeting the condition of *p* < 0.05 were defined as significantly enriched pathways.

KEGG pathway enrichment analysis with *p* < 0.05 as the threshold (Figure 6A) revealed that the highly enriched pathways in the turquoise module included ribosome (ko03010), aminoacyl-tRNA biosynthesis (ko00970), DNA replication (ko03030), pentose and glucuronate interconversions (ko00040), porphyrin metabolism (ko00860), fructose and mannose metabolism (ko00051), tryptophan metabolism (ko00380), mismatch repair (ko03430), starch and sucrose metabolism (ko00500), tyrosine metabolism (ko00350) and arginine and proline metabolism (ko00330). The highly enriched pathways in the blue module included glycerophospholipid metabolism (ko00564), vitamin B6 metabolism (ko00750), arginine and proline metabolism (ko00330), longevity-regulating pathway–multiple species (ko04213), pentose phosphate pathway (ko00030), and ether lipid metabolism (ko00565). The highly enriched pathways in the yellow module included arginine biosynthesis (ko00220), phenylalanine, tyrosine and tryptophan biosynthesis (ko00400), atrazine degradation (ko00791) and fatty acid degradation (ko00062).

Subsequently, networks were visualized for 124, 17, and 8 genes assigned to significantly enriched pathways in the turquoise, blue, and yellow modules, respectively (Figure 6B). Genes were ranked according to their Connectivity values within the corresponding network, and the most highly connected genes were identified as hub genes. The Connectivity values and rankings of the selected hub genes are provided in Table S4. In the turquoise module, the five most highly connected genes were *H6S33_000187* (cellulase), *H6S33_002256* (ribosomal protein S6e), *H6S33_010688* (ribosomal protein L5), *H6S33_011981* (tRNA synthetase class I), and *H6S33_012144* (pectate lyase). In the blue module, the five most highly connected genes were *H6S33_000837* (AAA domain/heat shock protein 78), *H6S33_001390* (dihydrodipicolinate synthetase family), *H6S33_003781* (adenosylmethionine decarboxylase), *H6S33_005711* (Ras family), and *H6S33_011492* (phosphatidylserine decarboxylase). In the yellow module, the four most highly connected genes were *H6S33_000437* (aminotransferase classes I and II), *H6S33_010159* (cytochrome P450), *H6S33_012547* (acyl-CoA dehydrogenase), and *H6S33_012643* (3-dehydroquinate synthase). These highly connected genes represent candidates for further investigation of the co-expression response associated with protein accumulation in *Morchella*.

**Figure 7:**
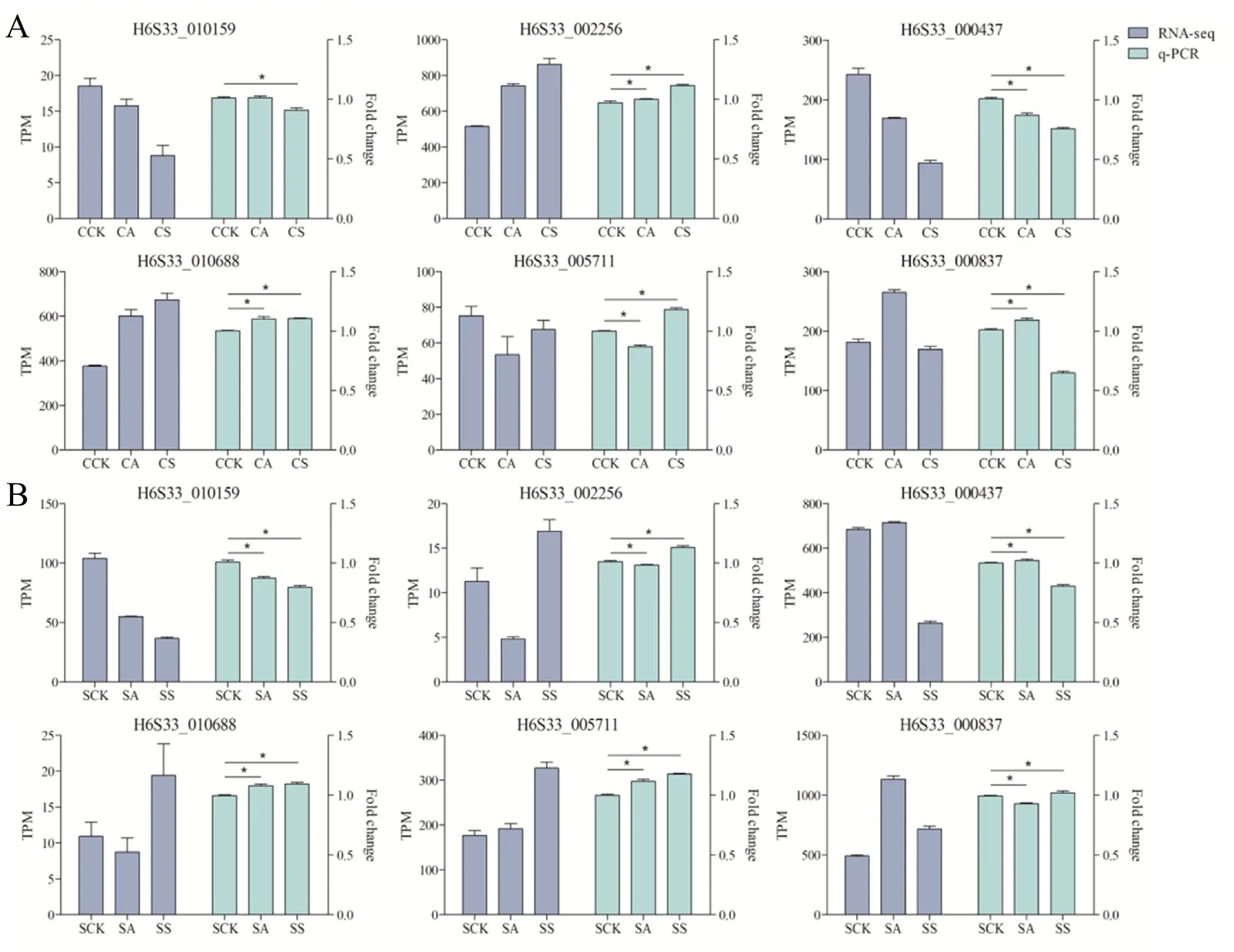
qRT-PCR validation of selected differentially expressed genes in the cap and stipe of *M. sextelata*. (**A**) Expression of six selected genes in the cap. (**B**) Expression of six selected genes in the stipe. The left and right Y-axes represent TPM and qRT-PCR fold change (2^−ΔΔCt^), respectively. Data are presented as mean ± SD of three biological replicates. Asterisks indicate significant differences between the treatments connected by horizontal lines (*p* < 0.05).

qRT-PCR analysis of six genes from each tissue broadly reproduced the concentration-dependent expression patterns obtained by RNA-seq (Figure 7). The selected genes in both the cap and stipe generally showed concordant directions of change across supplementation levels. Although the magnitude and peak treatment differed for several genes, the overall agreement between the two platforms supports the reliability of the transcriptomic data.

## 4. Discussion

As typical heterotrophic organisms, mushrooms secrete extracellular enzymes to degrade nutrients in the surrounding cultivation materials to obtain energy and metabolic building blocks, and consequently, the nutritional composition of mushroom fruiting bodies is strongly influenced by the composition and nutrient availability of the cultivation materials [33,34]. Nitrogen is a critical macronutrient that supports protein synthesis and directly affects mushroom yield, fruiting body development and nutritional quality [35,36]. Proteins present in the cultivation materials are degraded into amino acids by extracellular enzymes secreted by the mycelia, after which the amino acids are transported into fungal cells, assimilated, and used for protein synthesis [16,37]. The results of this study revealed that both soil application with 100 g/m^2^ soybean meal and nutrient-bag supplementation with 1% soybean meal significantly increased the protein content of *Morchella* fruiting bodies, consistent with previous reports that nitrogen-containing supplements can increase fruiting-body protein content [14,38]. In the cultivation of *Pleurotus ostreatus*, the addition of corn husk, oat husk, soybean nuggets and peanut shell in combination with soya as a nutritional supplement to the straw substrate significantly increased the protein content of fruiting bodies [39]. The use of urea (0.5% *w*/*w*) caused a 33.6% increase in the protein content of oyster mushrooms grown on sugarcane bagasse [40]. The supplementation of substrates with defatted pistachio meal and defatted almond meal resulted in a relatively high protein content of *Agaricus bisporus* [41]. Lentinus edodes cultivated in sawdust containing 40% wheat bran presented the best nutritional content [42].

Notably, different application methods for adding soybean meal had varying effects on the protein content of *Morchella*. In this study, compared with nutrient-bag supplementation, the soil application treatment was more effective at increasing the protein content of *Morchella*. Unlike most cultivated edible fungi, *Morchella* requires placement in an exogenous nutrient bag approximately 15 days after sowing to promote the colonization of mycelia inside the nutrient bag and facilitate the acquisition of the carbon and nitrogen sources necessary for growth. Soybean meal supplies protein-rich organic nutrients that may support mycelial growth and biomass accumulation [10], thereby promoting nitrogen absorption and assimilation and contributing to increased protein content in *Morchella* [35]. Although exogenous nutrient bags serve as the main carrier for supplying carbon and nitrogen sources during the growth of *Morchella*, the quality of the soil, as a core medium for mycelial colonization, nutrient exchange, and microbial interaction, also plays a crucial role in the nitrogen use efficiency and protein synthesis of *Morchella* [43]. The effect of soil-applied soybean meal on the protein content of *Morchella* was greater than that of nutrient-bag supplementation, which may be related to the slow release and efficient transformation of soybean meal-derived nutrients in the soil environment. Compared with the relatively restricted microenvironment within nutrient bags, soil contains a more diverse microbial community. The stronger effect of soil application may therefore be associated with the gradual transformation of soybean meal-derived nutrients in soil and the contribution of soil microorganisms to nitrogen availability during *Morchella* cultivation [11,44,45]. This interpretation is also consistent with the report that soil is an important source of nitrogen utilized by *Morchella* [9]. In addition, *Morchella* could grow normally in soils supplemented with 100 g/m^2^ soybean meal, as well as in nutrient bags supplemented with 1% and 10% soybean meal. However, soil application at 1000 g/m^2^ was accompanied by a significant reduction in *Morchella* yield and an increased occurrence of competing fungi. Excessive nutrient enrichment may disrupt the competitive balance of the soil microbial community and increase the risk of contamination by competing fungi [16].

Moreover, across all treatment groups, the protein content in the caps of *Morchella* was significantly greater than that in the stipes, indicating a tissue-specific pattern of protein accumulation. Although the caps and stipes of *Morchella* share the same genome, these distinct tissues exhibit substantial differences in gene expression, protein translation, metabolism, and even post-translational modification [46]. As the reproductive organ of *Morchella*, the protein synthesis process in the cap is closely associated with the production, development, and dispersal of spores [47]. In contrast, the stipe primarily functions in support and transportation; thus, its protein synthesis is more related to cell structure construction and substance transport [48]. Across all treatment groups, more DEGs were detected in the stipe than in the cap, indicating that the stipe is more sensitive to soybean meal supplementation at the transcriptional level. In the stipe, ribosome-related genes were predominantly downregulated under soil application, whereas genes associated with aminoacyl-tRNA biosynthesis were downregulated under nutrient-bag supplementation, indicating that the two application methods elicited distinct transcriptional responses in protein synthesis-related pathways. In the cap, the enrichment of the pentose phosphate pathway, glyoxylate and dicarboxylate metabolism, pentose and glucuronate interconversions, and fructose and mannose metabolism indicated that carbon metabolism was also involved in the response to soybean meal supplementation. Previous proteomic and metabolomic studies have similarly demonstrated marked differences in carbohydrate and energy metabolism between mushroom caps and stipes [46,48]. These findings suggest that soybean meal supplementation affected protein accumulation through tissue-specific transcriptional responses involving translation-related processes and carbon metabolism. The pathways, co-expression modules, and hub genes identified in this study provide candidate molecular components for further functional investigation.

The distinct transcriptional responses of the cap and stipe further indicate that these tissues may respond differently to soybean meal supplementation. Therefore, separate evaluation of the cap and stipe may provide a more accurate assessment of the effects of nutrient supplementation on the protein accumulation and nutritional composition of *Morchella* fruiting bodies. From a production perspective, the results indicate that the application method and dose of soybean meal jointly influenced protein accumulation and cultivation performance. Soil application at 100 g/m^2^ produced a greater increase in protein content than nutrient-bag supplementation, whereas increasing the soil application rate to 1000 g/m^2^ did not further improve cap protein content and was accompanied by reduced yield and an increased occurrence of competing fungi. Thus, moderate soil supplementation showed potential for improving fruiting-body protein content under the conditions of this study, whereas excessive supplementation increased cultivation risks. From a nutritional perspective, soybean meal supplementation increased the total protein content of *Morchella* fruiting bodies. However, the nutritional value of morels also depends on amino acid composition and other nutritional and bioactive constituents [4,5,49], while protein quality is additionally influenced by indispensable amino acid composition and digestibility [50]. Further studies should determine whether the increase in total protein content is accompanied by improvements in amino acid composition and protein digestibility. Together, these findings indicate that optimization of soybean meal application may help improve the protein content of *Morchella* while maintaining cultivation performance and limiting contamination risks.

## 5. Conclusions

This study demonstrates that the effects of soybean meal supplementation on *Morchella* were influenced by the amount supplied, the application method, and the tissue examined. Compared with supplementation through exogenous nutrient bags, soil application promoted greater protein accumulation, suggesting that the soil environment may facilitate the sustained availability and utilization of soybean meal-derived nutrients. The distinct responses of the cap and stipe further indicate that these tissues adopt different transcriptional and metabolic strategies when responding to an increased organic nitrogen supply. The co-expression modules, metabolic pathways, and hub genes identified in this study provide candidate molecular components linking nutrient supplementation with protein accumulation. These results provide reliable guidance for further optimizing the nutritional quality of *Morchella* and other edible fungi.

## Data Availability

The raw RNA-sequencing data generated in this study have been deposited in the NCBI Sequence Read Archive (SRA) under BioProject accession number PRJNA1274049 (https://www.ncbi.nlm.nih.gov/bioproject/PRJNA1274049, accessed on 28 August 2026).

## Supplementary Materials

The following supporting information can be downloaded at: https://www.mdpi.com/article/10.3390/jof12090660/s1, Table S1. Primers used in this study; Table S2. Summary of RNA-seq quality information; Table S3. Summary statistics of clean-read alignment to the reference genome; Table S4. Connectivity values and within-module rankings of hub genes in the turquoise, blue, and yellow WGCNA modules.
