# Supplementary material for: Transcriptome Analysis Reveals a Potential Response Mechanism of Morchella to Soybean Meal Addition
Source: J Fungi (Basel). 2026 Sep 2;12(9):660. doi: 10.3390/jof12090660 (PMC13608031; doi:10.3390/jof12090660)
Supplement: Supplementary file 1 [file jof-12-00660-s001.zip › jof-4479726-supplementary.pdf]

Supplementary Information

Table S1 primers used in this study.

Gene	F (5'-3')	R (5'-3')
Act	GTACCCTGGTATTGCCGACC	GGACGATGGAAGGACCACTC
H6S33_010159	CCCTTGACACTATCGCGTTG	CGATCCTCTCATCAGCAGGT
H6S33_002256	GGGACTGACTCGAAACCTCA	AAACCCTGCTTGTCGTTTCC
H6S33_000437	TCCGGCGTTTCTACACTTCT	GGAGCGCAATTCAGTACCAG
H6S33_010688	CGTGGTCTCAAGGTCAAGGA	TGGCAGGGTCGTACTTGATT
H6S33_005711	GAAGCTACGCGGTCAACAAT	GCCCAATTCCCTCGCTAAAG
H6S33_000837	AGGAAAGATTGCTGGACCGA	CCACCTTCCTTGACCCTGAT

Table S2 Summary of RNA-seq quality information.

Sample	Raw reads	Raw bases	Clean reads	Clean bases	Error rate (%)	Q20 (%)	Q30 (%)	GC (%)
CCK_1	42635652	6437983452	42327510	6356284116	0.0117	98.95	96.64	49.38
CCK_2	40616186	6133044086	40299746	6053919171	0.0117	98.94	96.61	49.4
CCK_3	44220068	6677230268	43887078	6583209953	0.0116	98.97	96.69	49.45
CA_1	42727138	6451797838	42310330	6363514453	0.0127	98.39	94.66	49.63
CA_2	41789372	6310195172	41478862	6231315958	0.0116	98.95	96.66	49.56
CA_3	40290940	6083931940	39974678	6019194362	0.0117	98.9	96.47	49.62
CS_1	44909936	6781400336	44576092	6694695656	0.0117	98.9	96.47	49.74
CS_2	42585808	6430457008	42256798	6357585388	0.0118	98.88	96.39	49.55
CS_3	47714824	7204938424	47355908	7124168703	0.0116	98.97	96.7	49.52
SCK_1	42212212	6374044012	41891490	6301685305	0.0118	98.87	96.39	49.25
SCK_2	44825704	6768681304	44466822	6690837074	0.0117	98.9	96.54	49.37
SCK_3	44194204	6673324804	43854324	6600290891	0.0118	98.86	96.39	49.39
SA_1	41917506	6329543406	41611172	6260795284	0.0117	98.91	96.51	49.44
SA_2	43726162	6602650462	43390676	6524182610	0.0117	98.92	96.58	49.4
SA_3	42787074	6460848174	42465622	6388103189	0.0117	98.89	96.48	49.42
SS_1	46268906	6986604806	45919120	6909849450	0.0117	98.9	96.53	49.29
SS_2	44368712	6699675512	44038756	6614220142	0.0117	98.89	96.5	49.24
SS_3	42820454	6465888554	42508134	6391194014	0.0117	98.91	96.55	49.29

Table S3 Summary statistics of clean-read alignment to the reference genome.

Sample	Total reads	Total mapped	Multiple mapped	Uniquely mapped
CCK_1	42327510	37877716(89.49%)	1087456(2.57%)	36790260(86.92%)
CCK_2	40299746	36233492(89.91%)	971840(2.41%)	35261652(87.5%)
CCK_3	43887078	39543435(90.1%)	1077742(2.46%)	38465693(87.65%)
CA_1	42310330	39252976(92.77%)	872583(2.06%)	38380393(90.71%)
CA_2	41478862	38544789(92.93%)	948244(2.29%)	37596545(90.64%)
CA_3	39974678	37167770(92.98%)	1065209(2.66%)	36102561(90.31%)
CS_1	44576092	40912743(91.78%)	1197845(2.69%)	39714898(89.09%)
CS_2	42256798	38898554(92.05%)	997789(2.36%)	37900765(89.69%)
CS_3	47355908	43632902(92.14%)	1028568(2.17%)	42604334(89.97%)

SCK_1	41891490	38697123(92.37%)	1204006(2.87%)	37493117(89.5%)
SCK_2	44466822	41006950(92.22%)	1041472(2.34%)	39965478(89.88%)
SCK_3	43854324	40454054(92.25%)	1202478(2.74%)	39251576(89.5%)
SA_1	41611172	39535906(95.01%)	1191708(2.86%)	38344198(92.15%)
SA_2	43390676	41203271(94.96%)	1109055(2.56%)	40094216(92.4%)
SA_3	42465622	40327554(94.97%)	1059313(2.49%)	39268241(92.47%)
SS_1	45919120	43805131(95.4%)	1064104(2.32%)	42741027(93.08%)
SS_2	44038756	42073758(95.54%)	1054619(2.39%)	41019139(93.14%)
SS_3	42508134	40474135(95.22%)	1447160(3.4%)	39026975(91.81%)

Table S4 Connectivity values and within-module rankings of hub genes in the turquoise, blue, and yellow WGCNA modules.

Module	Rank within module	Gene ID	Gene annotation	Connectivity
Turquoise	1	*H6S33_000187*	Cellulase	1182.48723
	2	*H6S33_011981*	tRNA synthetase class I	1176.30156
	3	*H6S33_002256*	Ribosomal protein S6e	1166.42908
	4	*H6S33_010688*	Ribosomal protein L5	1164.58751
	5	*H6S33_012144*	Pectate lyase	1163.57673
Blue	1	*H6S33_001390*	Dihydrodipicolinate synthetase family	1040.57843
	2	*H6S33_003781*	Adenosylmethionine decarboxylase	941.63483
	3	*H6S33_011492*	Phosphatidylserine decarboxylase	921.08658
	4	*H6S33_000837*	AAA domain/heat shock protein 78	830.72017
	5	*H6S33_005711*	Ras family	829.08248
Yellow	1	*H6S33_010159*	Cytochrome P450	756.17297
	2	*H6S33_012643*	3-Dehydroquinate synthase	669.39707
	3	*H6S33_000437*	Aminotransferase classes I and II	577.10244
	4	*H6S33_012547*	Acyl-CoA dehydrogenase	423.05101
